# Intron Retention as a Mode for RNA-Seq Data Analysis

**DOI:** 10.3389/fgene.2020.00586

**Published:** 2020-07-07

**Authors:** Jian-Tao Zheng, Cui-Xiang Lin, Zhao-Yu Fang, Hong-Dong Li

**Affiliations:** ^1^Hunan Provincial Key Lab on Bioinformatics, Center for Bioinformatics, School of Computer Science and Engineering, Central South University, Changsha, China; ^2^School of Mathematics and Statistics, Central South University, Changsha, China

**Keywords:** alternative splicing, intron retention, gene regulation, disease association, RNA-seq

## Abstract

Intron retention (IR) is an alternative splicing mode whereby introns, rather than being spliced out as usual, are retained in mature mRNAs. It was previously considered a consequence of mis-splicing and received very limited attention. Only recently has IR become of interest for transcriptomic data analysis owing to its recognized roles in gene expression regulation and associations with complex diseases. In this article, we first review the function of IR in regulating gene expression in a number of biological processes, such as neuron differentiation and activation of CD4^+^ T cells. Next, we briefly review its association with diseases, such as Alzheimer's disease and cancers. Then, we describe state-of-the-art methods for IR detection, including RNA-seq analysis tools IRFinder and iREAD, highlighting their underlying principles and discussing their advantages and limitations. Finally, we discuss the challenges for IR detection and potential ways in which IR detection methods could be improved.

## 1. Introduction

**Figure 1:**
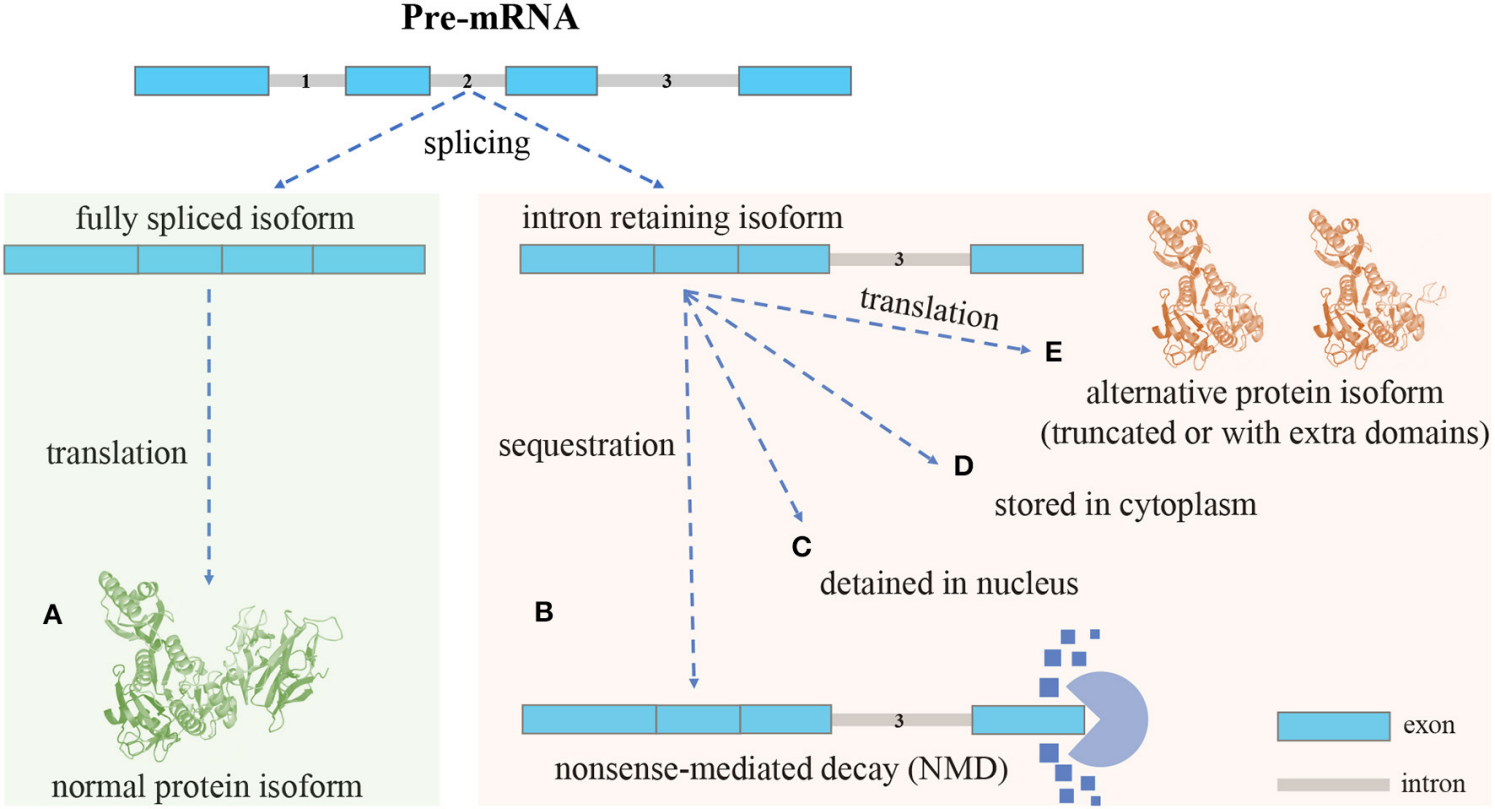
An overview of the intron retention (IR) mechanism: different isoforms can be produced from a single gene through AS. **(A)**, Isoforms with introns fully spliced are sent out of the nucleus for translation. Intron-retaining isoforms (IRIs) can be generated through IR (no intron retention): **(B)**, In most cases, the IRIs are degraded by the nonsense-mediated decay (NMD) pathway, the reason being that retained introns often contain premature termination codons (PTCs) that can trigger NMD (with intron retention): **(C)**, In some cases, the IRIs are detained in the nucleus, and in response to stimuli these IRIs can undergo further splicing to remove the retained intron, before being exported out of nucleus for translation (with intron retention): **(D)**, In the case of cytoplasmic splicing, IRIs are shuttled to the cytoplasm for preservation and may be subject to further splicing (with intron retention): **(E)**, In yet another case, IRIs escape from the NMD pathway and are translated into protein isoforms, which, compared with normal protein isoforms, are often truncated and may lose domains; however, it could also be that the alternative protein isoforms include extra domains formed by the amino acid sequences translated from retained introns (with intron retention).

Different mRNA splicing isoforms can be produced from pre-mRNA by skipping or joining coding/non-coding gene fragments, referred to as alternative splicing (AS) (Ner-Gaon et al., 2004). AS includes five major forms: exon skipping, intron retention (IR), mutually exclusive exons, alternative 5′ splice sites, and alternative 3′ splice sites. IR is the least understood form in mammalian cells (Sznajder et al., 2018; Monteuuis et al., 2019; Broseus and Ritchie, 2020). The process of IR is illustrated in Figure 1. In most cases, mature mRNA isoforms with introns fully spliced are exported out of the nucleus for translation (Cuenca-Bono et al., 2011; Palazzo et al., 2013). Because introns often contain premature termination codons (PTCs), intron-retaining isoforms (IRIs) are often rapidly degraded by the nonsense-mediated decay (NMD) pathway that is triggered by PTCs (Ge and Porse, 2014). IRIs may be retained in the nucleus or cytoplasm and be subject to further splicing in response to stimuli or stress (Naro et al., 2017). IRIs may also escape from the NMD pathway (Lykke-Andersen and Jensen, 2015) and be translated into protein isoforms that are often truncated (Lindeboom et al., 2016; Ottens and Gehring, 2016) and harmful to cells (Brady et al., 2017; Kanagasabai et al., 2017; Uzor et al., 2018; Mukherjee et al., 2019; Wang et al., 2019). Regarding the proportion of IRIs escaping from the NMD pathway, it has been shown that ~10% of human alternatively spliced nonsense-mediated decay (AS-NMD) transcripts are translated into truncated proteins (de Lima Morais and Harrison, 2010). Studies have shown that the truncated protein isoform may be shorter (i.e., have fewer domains) than or include extra domains over the normal protein isoform (Gontijo et al., 2011; Rekosh and Hammarskjold, 2018). As for the frequency of truncation, to the best of our knowledge, no estimates of the percentage of truncated proteins translated from IRIs seem to be available in the existing literature.

AS is a regulated process during gene expression (Koch, 2017). Since introns do not encode proteins, historically they were considered junk DNA as well as a burden on transcription and splicing (Wong et al., 2000; Roy and Irimia, 2008; Morris and Mattick, 2014; Parenteau and Elela, 2019). IR refers to an ineffective or inefficient splicing of introns that may have a negative impact on cells (Lim et al., 2011; Singh and Cooper, 2012; Wong et al., 2016). For example, IR of the *globin* gene will trigger NMD, which in turn affects red blood cell differentiation (Reimer and Neugebauer, 2018); IR generated an *Id3* isoform that limits the growth of smooth muscle cells during the formation of vascular disease (Forrest et al., 2004). IR is also associated with the development and maintenance of complex diseases. For example, many introns that are preferentially retained in primary cancers can be detected in the cytoplasm of cancer cells, and the abundant IRIs in cancer cells can increase the diversity of cancer cell transcriptomes (Dvinge and Bradley, 2015). In recent years, the transcriptome analysis of IR has received increasing attention.

Currently, the detection of IR is based on computational analysis of high-throughput RNA-seq data. In recent years, tools dedicated to IR detection have been developed, such as IRCall and IR classifier (Bai et al., 2015), Keep Me Around (KMA) (Pimentel et al., 2015a), intron Retention Analysis and Detector (iREAD) (Li et al., 2020), and IRFinder (Middleton et al., 2017). In addition, some tools originally designed to detect AS events can also be used to detect IR, such as mixture-of-isoforms (MISO) (Katz et al., 2010), multivariate analysis of transcript splicing (MATS) (Shen et al., 2012), replicate MATS (rMATS) (Shen et al., 2014), comprehensive alternative splicing hunting (CASH) (Wu et al., 2017), and DEXSeq (Anders et al., 2012). In recent years, deep learning-based AS detection methods have been developed, such as deep learning augmented RNA-seq analysis of transcript splicing (DARTS) (Zhang et al., 2019) and SpliceAI (Jaganathan et al., 2019).

In the following sections, we will review the association of IR with gene expression regulation and complex diseases. Last but not least, we will describe current computational approaches to IR detection and discuss their advantages and limitations.

## 2. Intron Retention in Gene Expression Regulation

IR plays an important role in regulating gene expression through triggering NMD (Wong et al., 2013; Ge and Porse, 2014). IRIs often contain PTCs (Braunschweig et al., 2014). The signal of a PTC can be recognized by the protein factors in the NMD pathway, and IRIs can thus be degraded by NMD. Consequently, IR leads to down-regulation of the isoform and of the protein products if translated (Ge and Porse, 2014). In this section we review some studies exploring the relationship between IR and the regulation of gene expression in different cell types, as well as studies investigating the relationship between IR and cell differentiation.

Some studies have found that IR is related to gene expression regulation in different types of cells. For example, Kienzle et al. (1999) suggested that retained introns can introduce a stop codon in an open reading frame or frameshift, which can contribute to gene expression regulation via premature termination of translation without changing the transcriptional activity. Taking the *EBNA-3* gene as an example, the presence of introns would effectively disrupt the translation process and thereby affect the expression of the *EBNA-3* protein, suggesting that IR may provide a means of fine-tuning the expression of the *EBNA-3* family gene in human B lymphocytes. Ni et al. (2016) found that the up-regulation of most genes in activated T cells was accompanied by a significant decrease in the level of IR. In their human and mouse CD4^+^ T cell validation experiments, 185 of 1,583 genes were mainly regulated by IR and were highly enriched in the proteasome pathway, revealing a novel post-transcriptional regulatory mechanism. This mechanism can help cells coordinate and respond quickly to extracellular stimuli, such as acute infections. Forrest et al. (2004) found that during the formation of vascular lesions in rats, an IRI called helix-loop-helix transcription factor Id3 (*Id3a*) was abnormally expressed in the early stage of lesion formation. Using the *Id3a*-specific antibody they developed, they found that the *Id3a* protein was induced to be translated in vascular lesions. This protein does not promote the growth of smooth muscle cells but stimulates their apoptosis and inhibits the production of endogenous *Id3a* isoforms.

Other studies have found IR to be associated with cell differentiation. By analyzing high-coverage poly(A)+ RNA-seq data, Braunschweig et al. (2014) found that the increase of IR during neuronal differentiation plays a major role in down-regulating gene expression. First, genes containing introns have higher retention rates in differentiated neurons than in murine embryonic stem cells and are significantly enriched in multiple Gene Ontology (GO) terms associated with the cell cycle. Second, the increase of IR reduces the mRNA expression of the *Ssrp1* gene during neuronal differentiation. Pimentel et al. (2015b) observed a dynamic increase of IR in late erythroblasts, indicating that IR explicitly regulates the differentiation process of erythroblasts. They also discovered many unique and extensive IR events during the differentiation of red blood cells. They inferred that IR is a multidimensional process that post-transcriptionally regulates multiple gene groups during normal erythropoiesis, and that its misregulation may be the cause of human disease. In the late phases of mammalian germ cell differentiation, the required transcripts must be synthesized and stored in advance (Paronetto and Sette, 2010). From observing the accumulation of the *ADAM3* protein, Naro et al. (2017) found that IRIs detained in the nucleus can regulate the use of transcripts.

In summary, there are a number of studies that show various ways in which IR can regulate gene/protein isoform production (Nilsen and Graveley, 2010; Floor and Doudna, 2016; Jacob and Smith, 2017), RNA stability, and translation efficiency (Thiele et al., 2006; Sterne-Weiler et al., 2013). These studies suggest that IR as a post-transcriptional splicing pattern plays an essential role in fine-tuning gene expression. (Mauger et al., 2016).

## 3. Intron Retention Is Associated With Complex Diseases

IR has been shown to be associated with complex diseases. For example, IR represents a mechanism (Mauger et al., 2016) and provides a sensitive and disease-specific diagnostic biomarker for neurodegenerative diseases (Jeromin and Bowser, 2017; Sznajder et al., 2018). In addition, IR was found to be widespread across a series of cancer transcriptomes (Dvinge and Bradley, 2015) and was thought to be related to tumor suppressor inactivation (Jung et al., 2015). We performed a literature survey and identified 60 papers (published after 2016) on the association of IR with diseases: 28 are on neurodegenerative diseases, 23 are on cancers, and the remaining are about other diseases, such as Duchenne muscular dystrophy (DMD), chronic lymphocytic leukemia (CLL), and myelodysplastic syndromes (MDS). Below, we will briefly review these studies on the association of IR with neurodegenerative diseases, cancers, and the other less-studied diseases.

With regard to neurodegenerative diseases, Xu et al. (2008) studied Alzheimer's disease (AD). A number of studies have shown that the apolipoprotein E4 (*apoE4*) isoform is associated with AD. In the primary neuron transfection experiment of Xu et al., they found that neuronal expression of the *apoE4* isoform was significantly higher when intron-3 was deleted from the genomic DNA structure and, conversely, significantly lower when intron-3 was inserted into the cDNA. This finding suggests that the retention/splicing of intron-3 controls the expression of the *apoE4* isoform in neurons, implying an association between IR and AD. Over-expression of the *peripherin* gene may lead to degeneration of motor neurons in transgenic mice. Xiao et al. (2008) identified the normal splicing variants of peripheral proteins and a novel transcript of peripheral proteins retaining introns 3 and 4. The IRI of the *peripherin* gene was found to be expressed at a low stoichiometric level. When the expression of IRI is up-regulated, it will lead to the aggregation of *peripherin*. This observation suggests that the abnormal splicing of peripheral protein in amyotrophic lateral sclerosis produces a splice isoform that is prone to aggregation.

Several studies have identified an association between IR and cancers. Zhang et al. (2014) used whole transcriptome sequencing data from five lung adenocarcinoma tissues and matched normal tissues to detect IR. A large number of IR events were found in both the tumor and the normal tissues, and 2,340 and 1,422 genes contained only tumor-specific and normal tissue-specific retention events, respectively. Subsequent functional analysis indicated that genes with tumor-specific retention include known lung cancer driver genes, such as *EGFR, ROS1*, and *RUNX1*, and are enriched in pathways that are important in carcinogenesis. IR in these genes causes frameshift, which generally invokes NMD and reduces the expression levels of mRNAs. These over-expressed or highly mutable driver genes may have a protective effect on patients. The work of Jung et al. (2015) demonstrated that IR is a mechanism leading to the inactivation of tumor suppressor genes. By analyzing the RNA sequencing and exome data from 1,812 cancer patients, they determined that at least 163 of the 900 splice-disrupted somatic exon single-nucleotide variants caused IR in an allele-specific manner and were enriched in tumor suppressor genes.

In particular, Dvinge and Bradley (2015) performed extensive experiments to analyze the association between IR and 16 cancers. By analyzing the genome-wide RNA splicing patterns of 805 matched tumor and control samples from 16 cancers, they found that abnormal RNA splicing occurs in the form of IR in cancers. The most common spliceosomal mutations, such as the specific missense changes of the *SF3B1, SRSF2*, and *U2AF1* proteins, are abundant in a variety of diseases, including MDS, lymphoid leukemia, and solid tumors of the lung, breast, pancreas, and eyes. They also used the transcriptomic data generated by the Cancer Genome Atlas project to identify large-scale differences in RNA splicing between the cancerous and control samples. In all the cancers except breast cancer, IRIs are up-regulated in the cancer samples, with the increase ranging from 2-fold (acute myeloid leukemia) to 40-fold (colon cancer) compared with control samples. Many introns that are preferentially retained in primary cancers are detectable in the cytoplasmic fractions of cancer cell lines. This finding suggests that IR is a common factor associated with tumorigenesis. Abundant IRIs in cancer cells may increase the diversity of cancer transcriptomes. Finally, through genome-wide quantitative analysis and unsupervised clustering analysis, Dvinge et al. confirmed that although some retained introns are shared by most cancer types, most are either present at a low frequency in multiple cancers or unique to primary cancers. For example, two adjacent introns in *FUS* were recurrently retained in multiple types of cancers, such as breast and colon cancers. Most introns (1,205 out of 1,767) were retained in a few samples of a particular cancer. The retained intron in *CDK10*, for example, was specific to and frequently retained in the colon cancer samples. Clustering results showed that cancers originating from similar tissues, such as the colon and rectum, have similar patterns of IR.

There are also studies on the association of IR with other less-studied diseases, such as DMD, CLL, and MDS. For example, the high-level retention of introns 40, 58, and 70 in DMD transcripts may be responsible for the lack of dystrophin expression in CRL-2061 cells, resulting in the elimination of the tumor suppressor activity of dystrophin (Niba et al., 2017). It was found that the *SF3B1* modulator sudemycin D6 (SD6) can effectively inhibit the growth of CLL cells and that IR in SD6-treated CLL cells increased significantly (Han et al., 2019). In the gene subset represented by *SF3B1*, the non-productive interaction between intron-terminal splice sites and decoy exons could prevent the excision of introns and was found to regulate a pivotal subset of IR events during erythroblast differentiation (Parra et al., 2018).

In recent years, splicing regulation therapy strategies have been developed and are currently being tested in clinical trials for a range of diseases (Scotti and Swanson, 2016; Di et al., 2019), including muscular dystrophy and motor neuron diseases. It is therefore increasingly important to understand the relationship between IR and diseases.

## 4. Methods for Intron Retention Detection

An important component of high-throughput sequencing, transcriptome sequencing technology (RNA-seq) is a useful tool in transcriptomics analysis (Conesa et al., 2016; Hrdlickova et al., 2017). RNA-seq data can be used to analyze transcriptome information, such as gene expression and splice sites (Vanichkina et al., 2018).

Currently, tools dedicated to IR detection are available (Bai et al., 2015; Pimentel et al., 2015a; Middleton et al., 2017; Li et al., 2020). Bai et al. (2015) developed IRcall (a ranking strategy) and IRclassifier (random forest classifiers) to detect IR events. IRcall integrates seven features—including gene expression, intron read counts, flanking exon read counts, and splice junctions—to calculate a joint score for IR events. The joint score can help to reduce false positive identification to a certain extent. IRclassifier constructs a training set based on the prediction of other IR detection methods and uses 21 features to characterize each intron; it then builds a random forest classifier to predict IR events. A limitation of these two methods is that the features used for model construction depend on the quality of the junction alignment tool (Bai et al., 2015). KMA (Pimentel et al., 2015a) is an IR detection pipeline that leverages existing isoform expression quantification tools. It can combine biological replicates to reduce the number of false positives. The isoform quantification and analysis of IR are performed in different software environments, which may be inconvenient (Li et al., 2020). IRFinder (Middleton et al., 2017) provides a complete pipeline for identifying IR events, including genome preparation, data preparation and quality control, IR quantification, and differential analysis. IRFinder quantifies IR in terms of splicing level and intronic abundance, where the *IR ratio* metric indicates the proportion of transcripts containing the intron of interest. IRFinder identifies IR candidates based on *IR ratio* and the number of intronic reads. iREAD (Li et al., 2020) uses the Shannon entropy to quantify the uniformity of the distribution of reads across the intron. To avoid ambiguity, only the independent intron that does not overlap with any exon of any gene is considered in iREAD. One limitation of iREAD is that it does not provide differential analysis.

In addition to the techniques above, methods for detecting AS can also be used to detect IR. For example, MISO (Katz et al., 2010) is a method for inferring isoform regulation from RNA-seq data. It models the generation process of reads in isoforms, considers all isoform expression levels in genes as random variables, and uses Markov chain Monte Carlo sampling to estimate the distribution of the variables. Therefore, MISO can estimate expression at both the AS event level and the whole mRNA isoform level. For differential analysis, MISO can perform comparison on only two samples (e.g., samples without replicates) (Wang et al., 2018). DEXSeq (Anders et al., 2012) is a method that was originally developed to detect exon usage. It can be used to detect IR if introns rather than exons are used as the genomic feature for calculating usage. DEXSeq integrates different methods to detect the deviation of reads of each exon, and the result is robust. Differential expression is performed for exon usage. MATS (Shen et al., 2012) uses a Bayesian statistical framework to detect differential AS from RNA-seq samples without replicates. rMATS (Shen et al., 2014) is an extended version of MATS and is capable of processing replicate samples. rMATS uses read counts that uniquely map to isoforms to estimate the exon inclusion level, and it takes into account both the uncertainty in individual samples and the variability between replicates. It is worth mentioning that rMATS adopts a flexible likelihood-ratio test, allowing users to define the threshold of inclusion level differences between groups. A limitation of rMATS is that it relies on known annotations of transcripts and has insufficient detection ability for novel AS events (Denti et al., 2018). In consideration of the currently incomplete transcript annotation, CASH (Wu et al., 2017) combines the annotated exon sites in the reference transcriptome and the novel splice site detected in RNA-seq data to reconstruct all splice sites for each gene. In this way, CASH has the potential to detect novel AS events (Carazo et al., 2019). Conventional quantification of exons (exon inclusion level) and isoforms (isoform ratio) depends on transcript models or predefined splicing events, which may be incomplete, partly because of the limitation of disease-specific abnormal transcripts (Li et al., 2018), for example. LeafCutter is an annotation-free method for quantifying both known and novel AS events based on exon-exon junction reads. It focuses on the intron removal rate rather than the exon inclusion rate (Li et al., 2018). The benefit of focusing on intron excision is that transcript annotation is not necessary and there is no need to estimate isoform or exon usage in complex splicing events. In brief, LeafCutter first defines introns that overlap and share the acceptor or donor splice site as intron clusters. The intron removal rate difference, quantified as ΔPSI of the intron cluster between samples, is used to find differentially excised introns. Although IR is not explicitly modeled by LeafCutter (Vaquero-Garcia et al., 2018), ΔPSI could reflect the possibility of IR.

Deep learning is a machine learning approach that has the ability to extract useful information or patterns from large numbers of samples. In recent years, deep learning has been introduced for AS analysis. For example, DARTS (Zhang et al., 2019) uses a large amount of sequencing data from public databases, such as ENCODE (Chi, 2016) and Roadmap (Romanoski et al., 2015) as input to the model. Then, the Bayesian hypothesis statistical test (BHT) is applied to obtain the training labels for each AS event. A deep neural network (DNN) is used to train all AS events. The prediction result of the DNN is fed into the BHT again to get the final prediction label for AS events including IR. SpliceAI (Jaganathan et al., 2019) uses a deep residual network to predict the splice sites of any pre-mRNA transcript sequence. The resulting splice sites can be used to infer whether IR has occurred. SpliceAI also explores the effects of gene mutations and exon and intron lengths on the splicing strength of splice sites.

## 5. Conclusion

The rapid development of high-throughput sequencing technology has enabled genome-wide detection of IR. Although significant progress has been made in this area, there are still challenges at present. First, current methods detect retained introns at the gene level instead of the isoform level. Identifying isoforms in which introns are retained is a question that remains to be resolved. Third-generation sequencing technologies, such as the PacBio single-molecule real time (SMRT) technology (Edge and Bansal, 2019), which can sequence the entire transcript, may help to address this challenge (Wu et al., 2017). Second, introns that are enriched in low-complexity and repetitive sequences may restrict the unique mapping of sequencing data (Broseus and Ritchie, 2020), and such introns if retained may be more difficult to detect. Third, there are currently no benchmark data available on retained introns, making it difficult to evaluate IR detection methods.

Current IR detection methods could be improved through integrating prior knowledge, selecting suitable thresholds for parameters, and so on. For prior knowledge, features, such as intron length, the distribution of the splicing regulatory elements, canonical or non-canonical status of splice sites, and splicing strength could be used as prior knowledge to improve IR detection (Mao et al., 2014; Cui et al., 2017; Kim et al., 2018; Zhang et al., 2018). For parameter thresholds, designing methods to incorporate sequence features and read coverage variations of introns to adaptively determine individual intron-specific optimal thresholds of parameters could be helpful for IR detection (Broseus and Ritchie, 2020). It is worth noting that any rigid thresholding may cause downstream analysis, such as GO enrichment to be heavily skewed toward genes with high expression (Young et al., 2010; Timmons et al., 2015). As an important mode of alternative splicing, IR is expected to advance our understanding of gene expression regulation and diseases from a new perspective (Wong et al., 2016; Jacob and Smith, 2017; Vanichkina et al., 2018; Monteuuis et al., 2019).

## Author Contributions

H-DL and C-XL conceived and wrote the manuscript. J-TZ wrote the manuscript. Z-YF wrote part of and reviewed the manuscript. All authors contributed to the article and approved the submitted version.

## Conflict of Interest

The authors declare that the research was conducted in the absence of any commercial or financial relationships that could be construed as a potential conflict of interest.
